# Enzyme Induced Biocementated Sand with High Strength at Low Carbonate Content

**DOI:** 10.1038/s41598-018-38361-1

**Published:** 2019-02-04

**Authors:** Abdullah Almajed, Hamed Khodadadi Tirkolaei, Edward Kavazanjian, Nasser Hamdan

**Affiliations:** 1 Assistant Professor, College of Civil Engineering, King Saud University, Riyadh, 11421 Saudi Arabia; 2 Postdoctoral Research Associate, Center for Bio-mediated and Bio-inspired Geotechnics (CBBG), Arizona State University, Tempe, AZ USA; 3 Regents’ Professor, Center for Bio-mediated and Bio-inspired Geotechnics (CBBG), Arizona State University, Tempe, AZ USA; 4 Assistant Research Professor, Center for Bio-mediated and Bio-inspired Geotechnics (CBBG), Arizona State University, Tempe, AZ USA

## Abstract

Specimens of silica sand treated via enzyme induced carbonate precipitation (EICP) showed surprisingly high strength at a relatively low carbonate content when non-fat powdered milk was included in the treatment solution. EICP is a biologically-based soil improvement technique that uses free urease enzyme to catalyze the hydrolysis of urea in an aqueous solution, producing carbonate ions and alkalinity that in the presence of calcium cations leads to precipitation of calcium carbonate. The strength achieved at less than 1.4% carbonate content via a single cycle of treatment was unprecedented compared to results reported in the literature from both EICP and microbially induced carbonate precipitation (MICP). Scanning electron microscope images show that in the specimens treated with the solution containing powdered milk the carbonate precipitate was concentrated at interparticle contacts. The impact of these results include reductions in the concentration of substrate and enzyme required to achieve a target compressive strength, reduction in the undesirable ammonium chloride by-product, and, depending on the desired strength, reduction in the number of cycles of EICP treatment. These advantages enhance the potential for development of a sustainable method of soil improvement via hydrolysis of urea.

## Introduction

### Background

Many geotechnical researchers are exploring the potential of calcium carbonate precipitation as a binder for granular soils. The technique that has been investigated most extensively by researchers is carbonate precipitation via hydrolysis of urea. This technique relies upon the enzyme urease to catalyze the hydrolysis of urea in an aqueous solution, forming carbonate ions and alkalinity that leads to calcium carbonate precipitation in presence of calcium ions. The most common method of carbonate precipitation via hydrolysis of urea described in the literature employs ureolytic microbes (microbes containing intra-cellular urease) as the source of urease and is referred to as microbially induced carbonate precipitation (MICP). Soil improvement via MICP has been a subject of research within the geotechnical community for the past 15 years^1–7^. More recently, the use of agriculturally-derived free urease enzyme for soil improvement, a process referred to as enzyme induced carbonate precipitation (EICP), has been investigated by a number of geotechnical researchers^8–14^.

**Figure 1:**
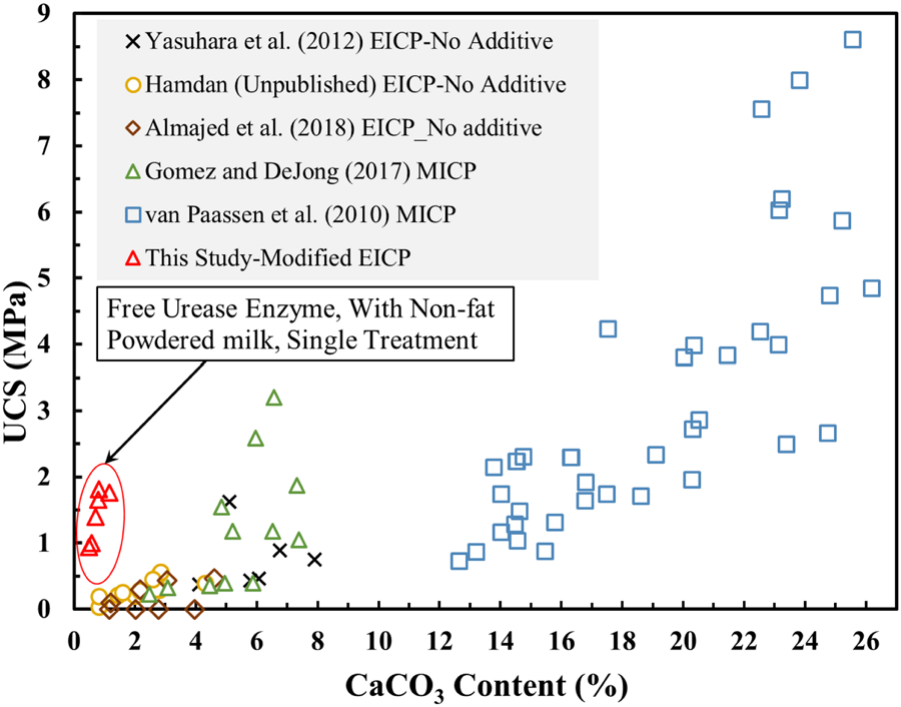
Relationship between unconfined compressive strength and calcium carbonate (CaCO_3_) content for specimens treated by EICP and MICP.

The typical measure of effectiveness used in studies on soil improvement via carbonate precipitation is the unconfined compressive strength of the soil. Figure 1 shows the relationship between precipitated calcium carbonate (CaCO_3_) content and unconfined compressive strength for soil treated via MICP and EICP based upon values reported in the literature. In all of these cases, a carbonate content in excess of 3% (w/w) and multiple cycles of treatment were required to achieve an unconfined compression strength in excess of 0.5 MPa.

### Study Rationale

We hypothesized that adding powdered milk into the EICP treatment solution would stabilize the enzyme and may also facilitate precipitation by providing nucleation points for carbonate precipitation and lowering the precipitation rate (which may be beneficial with respect to the morphology of the precipitate). Larsen *et al*.^15^ reported a ten-fold increase in calcium carbonate precipitation yield by using jack bean meal instead of purified urease enzyme. He attributed this increase in yield to protection of the urease proteins by other proteins, stabilizing the urease against environmental changes. Bachmeier *et al*.^16^ reported slower precipitation of calcium carbonate (potentially a beneficial effect with respect to crystal growth) with stabilized urease enzyme compared to non-stabilized free urease. Pretreatment using concentrated protein solutions such as Bovine serum albumin and milk have also been used to improve urease enzyme effectiveness with respect to precipitation efficiency^17,18^. Based upon these previous studies, we decided to employ non-fat milk powder, an inexpensive dairy product, in the EICP treatment solution. Casein proteins in milk can bind to the calcium ions in an EICP solution and salt-out, resulting in an aggregate or precipitate^19^. These precipitates may act as nucleation sites. Milk casein also has a marked chelating power versus calcium ions^20^ which may influence free calcium availability in the EICP solution and lower the precipitation rate.

## Materials and Methods

### EICP Treatment Solution

EICP treatment solutions were prepared by dissolving calcium chloride dihydrate (CaCl_2_.2H_2_O), urea, urease enzyme (with activity of ≈3500 U/g), and, in some cases, non-fat milk powder into 18.2 MΩ deionized (DI) water. Three different EICP treatment solutions were employed for soil treatment. Solution 1, referred to as the baseline EICP solution, was composed of 1.0 M urea, 0.67 M calcium chloride, and 3 g/l enzyme. These concentrations were selected based on our previous work^14^. Solution 2, referred to as the modified EICP solution, was composed of 1.0 M urea, 0.67 M calcium chloride, 3 g/l enzyme, and 4 g/l non-fat milk powder. Solution 3, referred to as the low concentration modified EICP solution, was composed of 0.37 M urea, 0.25 M calcium chloride, 0.85 g/l enzyme, and 4 g/l non-fat milk powder. The non-fat powdered milk contained 33% protein, less than 1.5% fat, less than 4% moisture, and 8.2% minerals.

### Soil Treatment

Ottawa 20/30 sand, a common silica sand used in geotechnical engineering research, (99% SiO_2_, mean grain size, D_50_, = 0.6 mm; coefficient of uniformity, C_u_, = 1.2; maximum void ratio, e_max_, = 0.742; minimum void ratio, e_min_, = 0.502; specific gravity of solids, G_s_, = 2.65; roundness, R, = 0.9; sphericity, S, = 0.9; regularity, ρ, = 0.9)^21^ was treated with the three different EICP solutions. Three specimens were prepared using each of the three EICP solutions. Test specimens were prepared by thoroughly mixing 350 g of sand with 75 ml (about one pore volume) of the EICP solution and then immediately placing the mixture into a 5.08 cm- (2 inch-) diameter acrylic column in three lifts. Each lift of sand was gently tamped so that the sand in the cylinder reached a final height of 10.16 cm (4 inches) above the base, corresponding to a relative density of 76%. Following densification of the sand by tamping, the treatment solution was always a few millimeters above the soil surface, indicating that the packed soil was in a near saturated condition. The top of each column was covered with aluminum foil in order to minimize loss of solution by evaporation. Each column was allowed to cure at room temperature (approximately 20 °C) for 3 days. After curing, the specimens were rinsed with about one pore volume of DI water and then oven-dried at 40 °C until no change in mass was observed. The dried specimens were subject to unconfined compression strength testing at a constant axial strain rate of 1.27 mm/minute.

### Carbonate Content Measurement

Gravimetric acid digestion was employed to measure the carbonate content of a portion of each specimen following unconfined compression testing. About 80–100 g (25–30%) of each specimen was soaked in a 4 M hydrochloric acid solution. The soaked specimen was then rinsed and dried. The mass difference before and after the acid digestion was considered to be the mass of calcium carbonate precipitated in the specimen. Mass of the precipitate over the mass of sand after digestion is reported in Table 1 as the carbonate content in each specimen.

### Microscale Identifications

X-Ray Diffraction (XRD) analysis was performed on intact pieces of selected specimens to identify the mineral crystal phases in each sample. The samples were ground using an agate mortar and pestle and powdered onto a standard glass slide for XRD analysis. Scanning electron microscopy (SEM) imaging was also performed on intact cemented chunks of material to visualize the morphology of the precipitates and the precipitation pattern in the soil. Energy dispersive X-ray (EDX) analysis was carried out in conjunction with SEM imaging to determine the elemental composition of the precipitates within each sample. The samples were coated with carbon prior to SEM/EDX analysis.

## Results and Discussions

The results of soil treatment in terms of unconfined compressive strength and carbonate content for specimens treated using the three different solutions are presented in Table 1. Comparing the unconfined compressive strength of the specimens treated using the baseline solution (Solution 1, no powdered milk) to the modified EICP solution (Solution 2, with powdered milk), adding powdered milk to the EICP solution resulted in unconfined compressive strength between 1.65 MPa and 1.82 MPa for the three specimens treated with the modified EICP solution while the specimens treated with the EICP solution that did not contain powdered milk had unconfined compressive strengths of between 0.12 MPa and 0.16 MPa at similar carbonate content. The dramatic increase in strength for the specimens treated with the modified EICP solution was unexpected.

**Table 1:** UCS results and carbonate content in the specimens treated with three different EICP solutions.

EICP Solution	Test No.	Peak Strength^*^ (kPa)	Mean Strength (kPa) ± STDV.	CaCO_3_ (%)	Mean CaCO_3_ (%) ± STDV.
Solution 1 (Baseline EICP)	1	133	138 ± 18	1.63	1.20 ± 0.37
	2	158		0.98	
	3	124		0.99	
Solution 2 (Modified EICP)	1	1817	1745 ± 83	0.82	0.93 ± 0.21
	2	1654		0.79	
	3	1763		1.17	
Solution 3 (Modified EICP)	1	1000	1112 ± 247	0.57	0.59 ± 0.20
	2	1396		0.71	
	3	941		0.49	

*Peak strength is the maximum axial compressive stress that each specimen can withstand during a UCS test.

Comparing the specimens treated with Solution 2 (the modified EICP solution) to those treated with Solution 3 (the low concentration modified EICP solution), lowering the concentration of urea and calcium chloride by 62.5% (from 1.0 M urea and 0.67 M CaCl_2_ to 0.37 M urea and 0.25 M CaCl_2_) and the concentration of enzyme by about 72% (from 3 g/l to 0.85 g/l) led to a reduction of approximately 35% in the unconfined compressive strength of the treated specimens. The average unconfined compressive strength decreased from approximately 1.7 MPa for the three specimens treated with the modified EICP solution to an average unconfined compressive strength of approximately 1.1 MPa for the specimens treated with the low concentration modified EICP solution. Note that the unconfined compressive strength of the specimens treated with the low-concentration modified EICP solution (Solution 3, which contained powdered milk) was still an order of magnitude greater than the unconfined compressive strength of the specimens treated with the higher concentration solution that did not contain powdered milk (Solution 1, the baseline solution).

The theoretical maximum carbonate content was approximately 1.4% for the specimens treated with Solutions 1 and 2 (the higher concentration solution) and was about 0.5% for the specimens treated with Solution 3 (the lower concentration solution). For the three specimens treated with Solution 1, the measured carbonate content varied from 30% less to 16% more than the theoretical maximum. For the three specimens treated with Solution 2, the measured carbonate content varied from 41% less to 16% less than the theoretical maximum. For the three specimens treated with Solution 3, the measured carbonate content varied from 2% less to 42% more than the theoretical maximum. Variations in carbonate content may be attributed in part to non-uniform distribution of the precipitate within each specimen, as only a portion of each specimen was used for acid digestion. Loss of precipitates suspended in the pores or loosely attached to the sand particles due to rinsing at the end of the treatment process may also result in a measured carbonate content less than the amount of carbonate precipitated from the treatment solution^14^. Furthermore, small human errors can be magnified in the gravimetric acid digestion method in specimens with a relatively low carbonate content.

**Figure 2:**
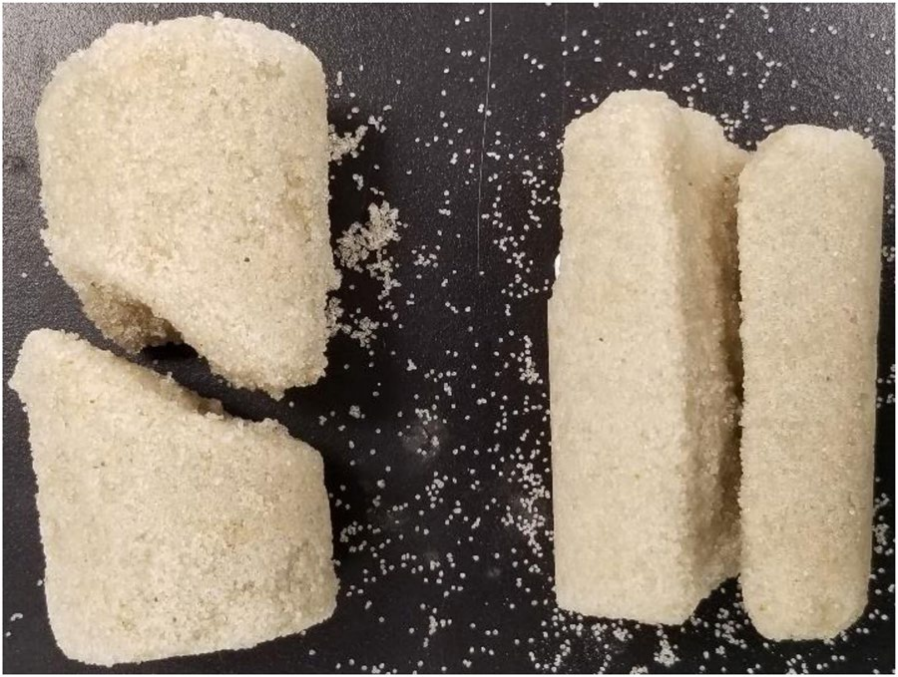
Failure patterns of EICP-treated specimens: shear failure of specimens treated with baseline solution (no powdered milk) (left); and tensile splitting of specimens treated with modified solution (with powdered milk) (right).

**Figure 3:**
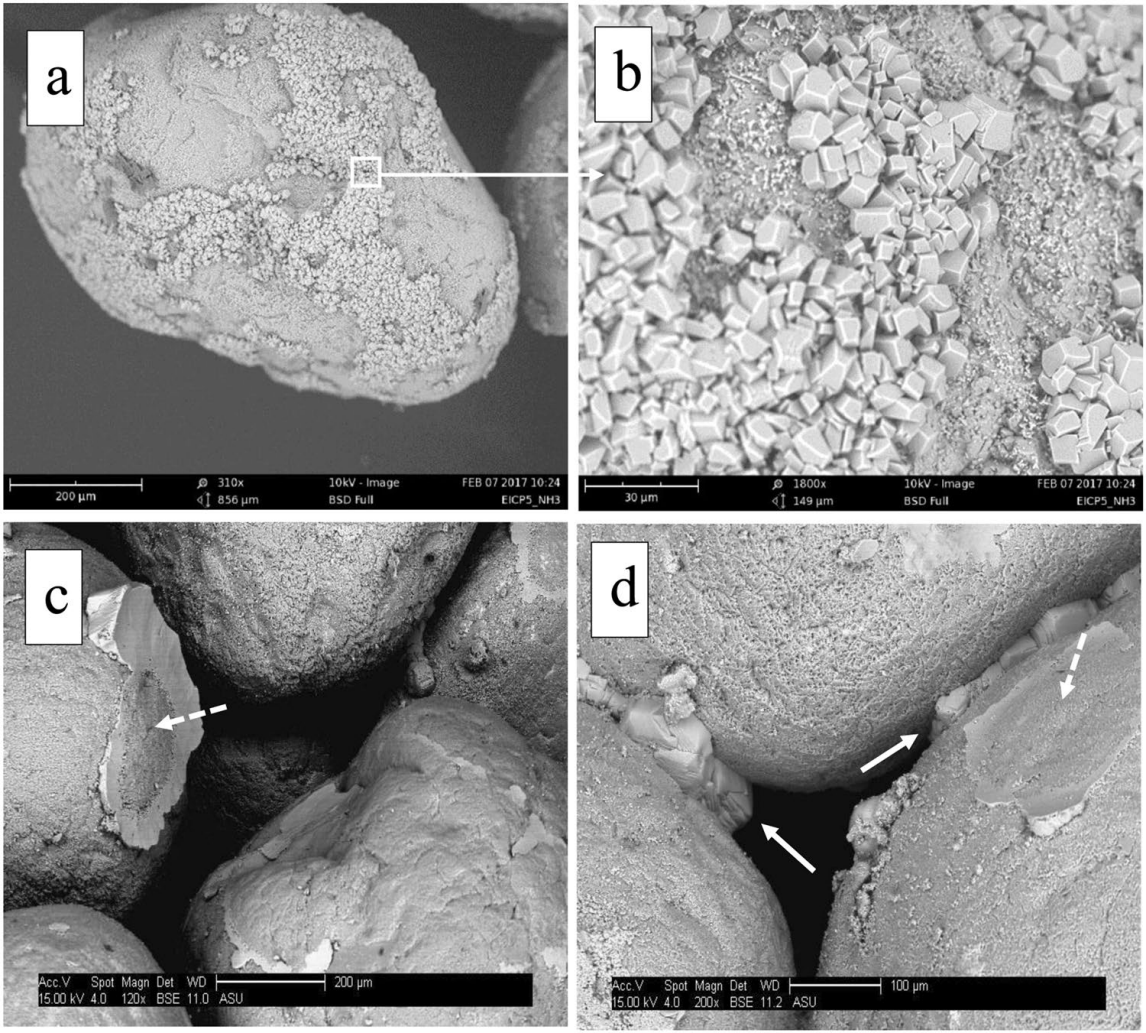
SEM images of: (**a**,**b**) soil particles treated using the baseline EICP solution (no powdered milk) showing relatively small calcite crystals cladding the particle surface and (**c**,**d**) soil particles treated using the modified solution (with powdered milk) showing relatively large calcite crystals focused at inter-particle contacts (solid arrows show inter-particle bonds; dashed arrows point to broken inter-particle bonds).

**Figure 4:**
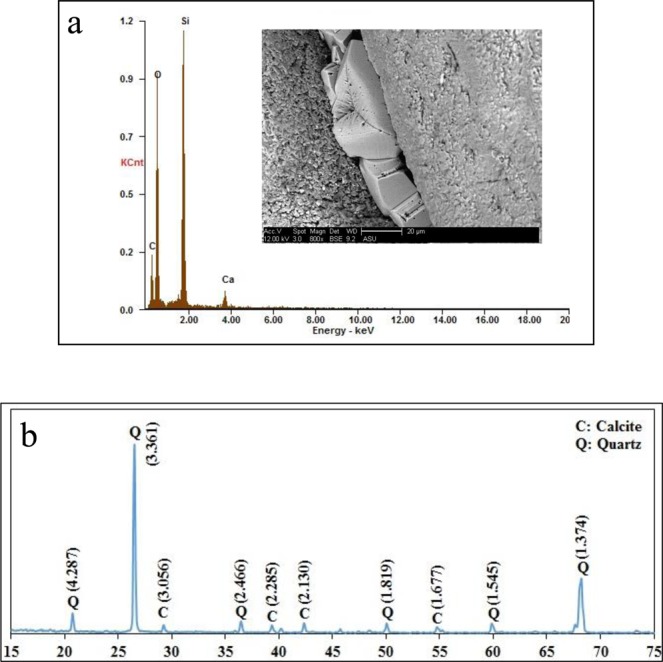
(**a**) SEM image of rhombohedral calcite crystals at an interparticle contact and the results of EDX analysis of this region confirming the presence of calcium carbonate and silica; and (**b**) XRD spectrum showing peaks corresponding to the calcite crystal phase and quartz sand. The values in the prenteces are d spacing value in Å.

Observations of the failure pattern of the specimens following unconfined compressive loading indicated that the specimens treated using the Solution 2 and Solution 3 failed by tensile splitting whereas the specimens treated with the Solution 1 failed in shear. Figure 2 shows the different failure pattern of the Specimen 1 and Specimen 2. SEM images, presented in Fig. 3, show a difference in precipitation patterns between the specimens treated with and without powdered milk in the EICP treatment solution. For the specimens treated using the solution that did not contain powdered milk, the precipitated carbonate appears to be in the form of relatively small crystals distributed over the surface of the sand particle. When powdered milk was added into EICP solution, it appears that relatively large calcite crystals formed and that the precipitation was focused mainly at inter-particle contacts. The pattern of precipitation is believed to be a major contributor to the increase strength of the specimens prepared with an EICP solution containing powdered milk compared to specimens treated with an EICP solution that did not contain powdered milk. The results of EDX and XRD testing, presented in Fig. 4, together confirmed precipitation of calcium carbonate in the calcite phase.

**Figure 5:**
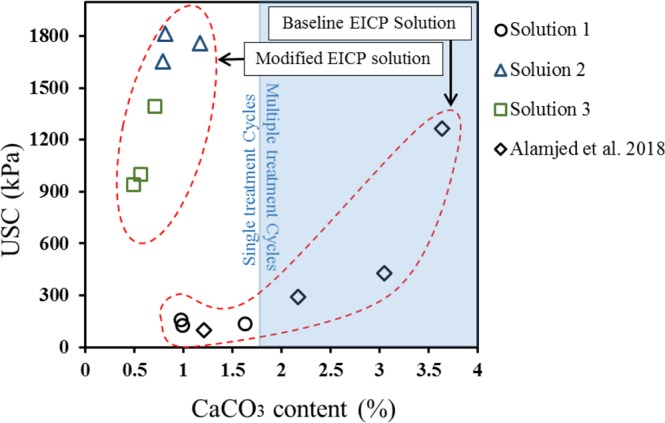
Unconfined compressive strength versus calcium carbonate (CaCO_3_) content for specimens treated by EICP.

The surprising unconfined compressive strength data from the tests reported herein wherein powdered milk was used in the treatment solution are included on Fig. 1. Figure 5 presents an expanded view of the unconfined compressive strength versus carbonate content relationship among the tests from this study. Figure 5 also includes the results of tests reported by Almajed *et al*.^14^ on soils treated using treatment Solution 1 (the solution without powdered milk) in which specimens were subject to multiple cycles of treatment to generate carbonate contents greater than 2%. Note that in all of the previous studies on carbonate precipitation, including studies employing both MICP and EICP, a minimum of at least 3% precipitation and multiple treatment cycles were required to achieve a strength equal to or greater than 0.5 MPa.

Understanding the surprising effects of adding milk powder to the EICP solution requires more detailed investigations. However, we can speculate on some of the effects of adding milk powder based on the chemistry of milk. The relatively large calcite crystals precipitated in the specimens treated with the modified (milk-containing) EICP solutions may be due to the slower rate of precipitation. Molecular interactions between urease enzyme and milk proteins can reduce the accessibility of active sites on the enzyme to urea and consequently lower the precipitation rate, consistent with the findings of Bachmeier *et al*.^16^. Casein in the milk may also act as a chelating agent reducing the precipitation rate. In addition, casein might precipitate and provide nucleation sites that favor calcite crystal formation and growth.

## Conclusion

The test results presented in this paper show that the addition of non-fat powdered milk to an EICP treatment solution resulted in surprisingly high unconfined compressive strength of Ottawa 20/30 silica sand at relatively low carbonate content compared to previous treatment strategies for precipitation of calcium carbonate. The strength that was achieved at carbonate contents of less than 1.4% and via a single cycle of treatment in these tests is unprecedented compared to results reported in the literature from from both MICP and EICP treatment. Samples treated with an EICP solution that included powdered milk yielded unconfined compressive strengths of 1.6 to 1.8 MPa with a theoretical maximum carbonate content of 1.4% and with a measured carbonate content of less than 1.0% for the moodified EICP solution used in the tests reported herein. SEM imaging and EDX testing revealed that the primary polymorph of the precipitates in the specimens formed using the EICP solutions containing powdered milk was calcite and that the calcite precipitation was focused at the contact points between particles. This pattern of precipitation is believed to be a major contributor to the increase strength compared to specimens treated without adding powdered milk to the EICP solution.

Importantly, these results show that the unconfined compressive strength of soils improved by carbonate precipitation depends not only on the amount of precipitated carbonate but also on the pattern of precipitation. A relatively high strength can be achieved at a relatively low carbonate content if the pattern and morphology of the precipitates are favorable. The significant advantages of the high strength at low carbonate content achieved by adding powdered milk to the EICP treatment solution as observed in this testing program are reductions in the concentration of substrate and enzyme required to achieve a target compressive strength, reduction in production of the undesirable ammonium chloride by-product generated by the hydrolysis of urea, and, depending on the desired strength, reduction in the number of cycles and duration of treatment. These advantages enhance the potential for development of a practical, cost effective, and sustainable method of soil improvement via hydrolysis of urea.

## Data Availability

The data that support the findings of this study are available from the corresponding author upon request.
